# On-Demand Sensor Node Wake-Up Using Solar Panels and Visible Light Communication

**DOI:** 10.3390/s16030418

**Published:** 2016-03-22

**Authors:** Carolina Carrascal, Ilker Demirkol, Josep Paradells

**Affiliations:** 1 Department of Telematics Engineering, Universitat Politecnica de Catalunya, Barcelona 08034, Spain; carolina.andrea.carrascal@alu-etsetb.upc.edu (C.C.); josep.paradells@entel.upc.edu (J.P.); 2 Fundacio i2CAT, Barcelona 08034, Spain

**Keywords:** wake-up radio, wake-up receiver, Visible Light Communication (VLC), solar panel, energy harvesting

## Abstract

To significantly reduce, or eliminate completely, the energy waste caused by the standby (idle) mode of wireless sensor nodes, we propose a novel on-demand wake-up system, which allows the nodes to be put into sleep mode unless their activation is truly necessary. Although there have been many studies proposing RF-based wake-up radio systems, in this work, we develop the first visible light communication (VLC)-based wake-up system. The developed system can extend the existing VLC systems and can be exploited to derive new application areas such as VLC tags. The system uses an off-the-shell indoor solar panel as receptor device of the wake-up signal as well as for energy harvesting purposes, through which it is able to harvest enough energy for its autonomous work. The design, implementation details and the experimental evaluation results are presented, which include flickering characterization and wake-up range evaluations. The results show that the developed system achieve reasonable wake-up distances for indoor environments, mainly where the use of VLC systems are considered.

## 1. Introduction

To achieve longer battery life of wireless sensor nodes, a common method employed is duty-cycling. In duty-cycling, the wireless nodes listen to the channel for potential incoming communications turning their radios on periodically, and remaining in sleep mode the rest of the time. It has been shown that this approach can better preserve energy, however, it causes energy waste when the device wakes up and there is no data to transmit or receive. A more efficient solution consists of on-demand communication, *i.e.*, totally asynchronous and rendezvous-less communication, achieved by wake-up radios. Wake-up radios are small and low- or no-power devices that activate the wireless sensor nodes when an external wake-up call occurs. The use of a wake-up receiver permits the wireless nodes to remain in sleep mode as long as possible. It has been shown that wake-up radio enables a more energy-efficient approach compared to duty-cycling mechanisms, due to the elimination of the useless idle periods of the node [1].

The harvesting of energy from the environment, however, becomes an interesting option in wake-up receiver devices in the path towards an energy-autonomous communication system. A relatively less explored energy source for harvesting energy is indoor lighting, although many wireless communication applications happen in indoor environments. With current indoor solar panel technology, the amount of power harvested is limited (e.g., less than 90 μW by the solar panel evaluated in this study for a light intensity of 200 lux). However this amount of power can be used for very low power communication devices such as wake-up receivers, e.g., less than 30 μW is required by the system proposed in this study. In this work, we propose the use of solar panel to develop a wake-up communication system using Visible Light Communication (VLC) as communication channel of the wake-up signal. Our goal is to create a wake-up system where an indoor solar panel is used both as the receptor of the VLC signal and also as energy harvester. To the best of our knowledge, this is the first wake-up communication system that exclusively uses light harvesting, paving the way to a new research direction.

In a preliminary work, we presented the first stage in the development of this system [2], where we described the general wake-up system and the components used. In this work we describe the complete system and use the indoor solar panel also for harvesting the energy for the wake-up device operation. This novel wake-up communication system will then allow: (i) the use of light as a wireless channel for the wake-up signal; (ii) to put the wireless sensor nodes to deep sleep mode to conserve energy, until it is really necessary to enter in active mode; (iii) to enable the wake-up device to work through an addressable wake-up, *i.e.*, waking up only the wireless nodes that are destined; (iv) to contribute the research direction of battery-less wake-up receiver by using the solar panel both for charging and communication purposes; and (v) to enable novel applications such as light-driven localization, asset control, *etc*.

To devise a feasible and efficient solution, we evaluated different system component alternatives through physical experiments. Specifically, we investigated the performance trade-offs brought by the solar panel size, the use of a photodiode as a communication receptor alternative, and the choice of correlator for addressable wake-ups. The characterization of the off-the-shelf indoor solar panel chosen is performed for its frequency response and current, voltage and power generated for different load values, based on which operational frequency of the system is chosen along with the wake-up receiver circuit design.

A low-cost and flexible VLC wake-up transmitter solution is developed through a small form-factor, wirelessly controllable VLC transmitter module devised in this paper. Through physical experiments, the complete system performance is evaluated in terms of wake-up probabilities achieved per transmitter-receiver distances, resiliency of the system to interference from fluorescent light, which is one of the most common lighting technology used in the office environments. A crucial challenge of VLC is flickering of the light, especially for low data-rate communication such as the one targeted in this work. The flickering characterization of the system is presented, along with the evaluation of possible mitigation solutions. Finally, different system configurations such as bit duration settings, use of capacitors for energy storage are assessed for detailed system performance evaluations.

With the final configuration proposed, a 14 m wake-up distance is achieved in scenarios without interferences. Moreover, with the proposed flickering mitigation techniques, a 7 m wake-up distance is achieved with no noticeable flickering. According to the experiment results, the developed system provides feasible operational distance for many indoor applications, such as asset control, tracking and monitoring of devices.

The rest of the document is structured as follows: Section 2 gives an overview of the state of art of related work. Section 3 presents the system design and the implementation details of the wake-up system. In Section 4, we present the experimental results. Finally, Section 5 concludes the paper and presents potential future work.

## 2. Related Work

The use of visible light as medium of communication is known as Visible Light Communication (VLC). Current efforts in the IEEE 802.15.7 study group [3] includes low-rate communication, which is one focus of this current paper, specifically using VLC as a channel to wake up a sleeping wireless node. In [4], the use of a solar cell as a simultaneous Visible Light Communication and optical energy receiver is demonstrated. In that work, a characterization of a solar panel is made and a distance of 40 cm is achieved with a VLC data rate of 3 Kbps. The experiment is set with the use of a frequency generator at transmitter side and a solar cell and oscilloscope at receiver side. In this work, we propose a complete system including transmitter and receiver that can be easily implemented as part of a wirelessly controlled lighting infrastructure, with a wake-up distance of up to 14 m.

Wireless wake-up system studies in the literature mainly focus on radio frequency (RF) triggered approaches. There are few studies in the literature where a different communication mode other than RF is used for wake-up communication. A studied mode is Free-Space Optical (FSO) communication, which covers both the VLC and the infrared (IR) spectrum. In [5], a device that uses a FSO communication as a wake-up channel is described. The device is designed to be used within indoor networks, and requires line of sight with a highly directional communication between transmitter and receiver. The wake-up receiver is equipped with photodiodes, which are used as signal receivers. The transceiver in a receive mode has a power consumption of 317 μW. This proposal reaches a communication range of 15 m with a transmitter with a power consumption of 16.5 mW. Compared to [5], the VLC wake-up receiver presented in this paper includes the following novel aspects: (i) the use of an indoor solar panel as receptor of the signal, which we show to perform better than photodiodes for wake-up purposes; and (ii) the harvesting of light energy for feeding purposes.

In [6], an active wake-up receiver that works with infrared signals is proposed. Depending on the transmitter signal intensity, the operational range of wake-up signal reaches up to 30 m. This device has a consumption of 12 μW while waiting for an incoming signal. Ambient light is found to saturate the receiver and increase the power consumption. In the case of our implementation, we exploit the lighting infrastructure for lighting, communication and energy harvesting purposes.

In [7], a 695 pW standby power optical wake-up receiver for wireless sensor nodes is introduced. The receiver consumes 695 pW in standby mode and in active mode 140 pJ/bit at 91 bps. A pulse width modulated communication encoding scheme is used, and chip-ID masking enables selective batch-programming and synchronization of multiple sensor nodes. Using a laser at transmitter side, a distance of 50 m is reached. Using a 3 W LED focused it reach a distance of 6 m. In [8], a 4.4 μW wake-up receiver using ultrasonic communication is presented. The device achieves a distance of up to 8.6 m for a data rate of 0.25 kb/s with −18 dBm signal power going into the transmit transducer.

In our work, we use solar panel which paves way to a wake-up receiver of ultra-low power consumption. A comparison of the different wake-up communication modes is given in Table 1. For comparison purposes, the details of a recent study on RF-based wake-up receiver [9] is also included.

## 3. Solar Panel and VLC Based Wake-Up Communication System: Design and Implementation

### 3.1. System Design

Wake-up communication systems mainly consist of four components: Wake-up transmitter (WuTx), wake-up receiver (WuRx), the data communication modules attached to WuTx and to the WuRx. In certain implementations, WuTx and/or WuRx is part of the data communication module as in [1]. WuTx and WuRx hardware might also be implemented jointly into a wake-up transceiver. In this work, our implementation will include the four components, implementing separate WuTx and WuRx components.

**Figure 1:**
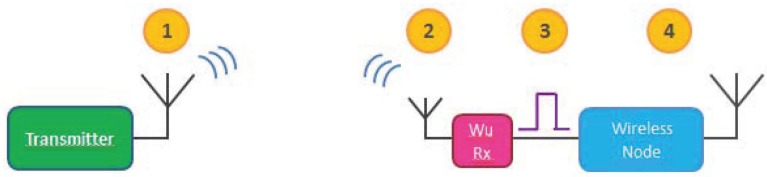
Wake-up communication system operation.

Figure 1 illustrates the wake-up communication system operation. First, the transmitter sends a signal intended for the wireless node (Step 1). The wake-up receiver is constantly sensing the channel (Step 2) and upon reception of a signal, it demodulates the data within the signal and compares the address included in the data to its address. If the addresses match, the WuRx triggers a pulse through an interrupt pin (Step 3) in order to wake up the wireless node attached, notifying the node about an incoming communication. Finally, the wireless node wakes up (Step 4) and is ready to perform the data communication. The use of a wake-up device permits the nodes to remain in an energy conserving stage as long as possible, where the node’s MCU/CPU put to a deep-sleep mode to be only triggered by an external interrupt and the main data transceiver is switched off. To achieve an energy-efficient operation with a low form factor overhead, the wake-up receivers are required to have a small footprint with an ultra-low or no power consumption.

**Figure 2:**
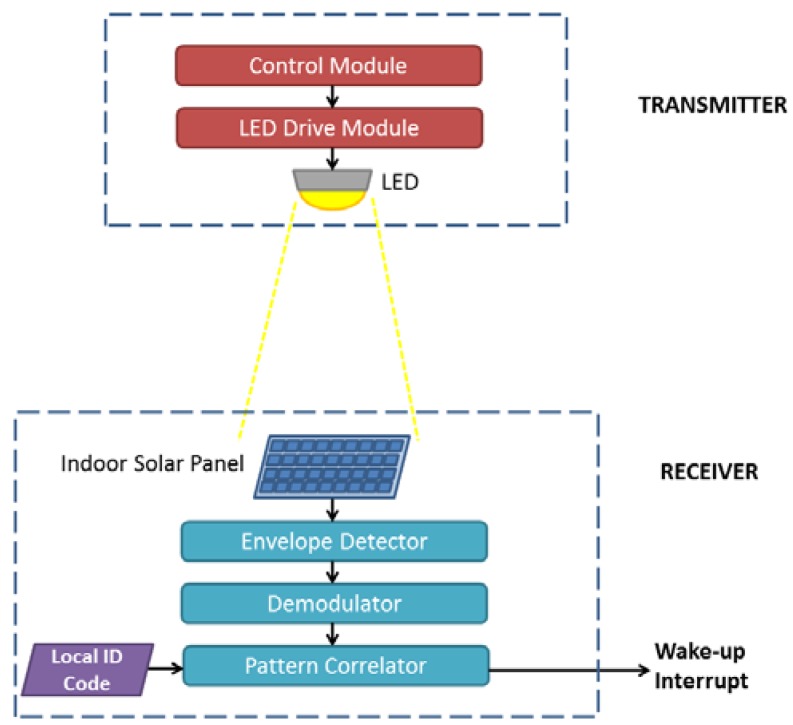
Global system view of the solar-panel-based VLC wake-up system.

In this study, we target to use visible light as the communication medium (Step 1), while an indoor solar-panel is used as the receptor of wake-up signal (Step 2). To realize the wake-up communication system targeted, the required system components are presented in Figure 2. At the transmitter side, the main components are a Control Module with a logic for the modulation of the light, a LED Drive Module for varying the amount of current passing through the LED, and a LED used for indoor lighting purposes.

The receiver side is comprised of an indoor solar panel, which works as both a receptor and as an energy harvester. The other components of the receiver are the modules in charge of the signal detection, its demodulation and correlation, *i.e.*, comparison with the local ID code (address). Once the wake-up interrupt signal is triggered and consequently the wireless node is awoken, the communication between transmitter and receiver can be performed using the main data communication interface of the nodes. The design presented in this work can be easily adapted to work with different application requirements. For example, by adding a secondary reception path, once the receiver node is awoken, the system can support downlink data communication through the use of LED and indoor solar panel.

### 3.2. System Implementation Details

#### 3.2.1. WuRx Receptor: Indoor Solar Panel

For the design of our system, the choice, and therefore the characterization, of the indoor solar panel is crucial since this device has two important tasks: To harvest the energy to feed the WuRx device and to receive the VLC signal. Note that, the amount of power that can be harvested by the indoor solar panel is a determining factor for the selection of the other receiver components.

After preliminary studies, we selected an off-the-shelf indoor solar panel for wide-range of availability and for its good operational performance as indicated in the following. The chosen solar panel is SC3726I-8-1 (Blue Solar Company, Dongguan, China), which is an amorphous silicon solar cell with a rectangular shape of size 3.6 cm × 2.6 cm and of thickness 0.1 cm. The open circuit voltage of the solar panel is measured to be around 5.6 V and its short circuit current is 34.6 μA. In order to characterize the performance of the chosen indoor solar panel and compare it to other alternatives, four tests were conducted: (i) current, voltage and power characteristics for different resistor values; (ii) frequency response characterization of the indoor solar panel for different VLC modulation frequencies; (iii) a performance comparison with a photodiode used as a VLC receiver; and (iv) its performance comparison with an off-the-shelf indoor solar panel with a larger surface area.

**Figure 3:**
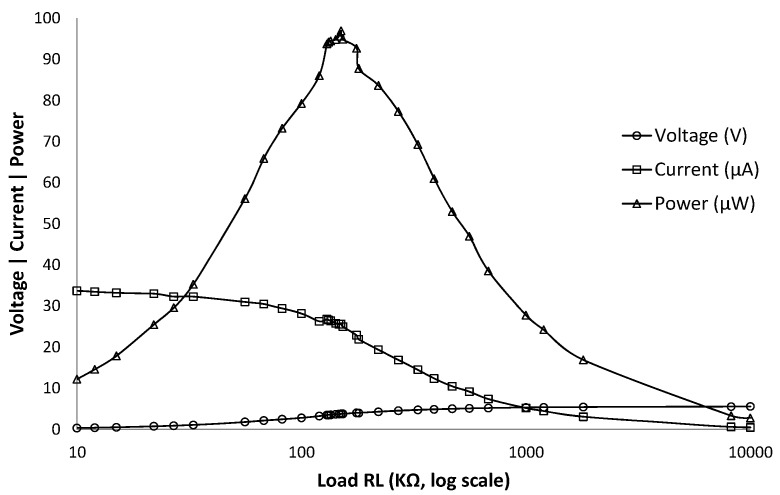
Current, voltage and power characteristics of indoor solar panel evaluated for different resistor values.

The first test lets us find out the maximum power of the solar panel in an indoor environment with a measured light intensity of 200 lux. This light intensity value is chosen as it corresponds to a typical indoor environment light intensity level [10]. As shown in Figure 3, different resistor values were tested and the maximum power is found to be 96.9 μW with a load of 150 KΩ. For the second test, the goal was to identify the response of the indoor solar panel to different frequencies to characterize and choose operational frequency for VLC communication. In the test performed, light modulated with different frequencies were projected to the panel. The frequency response of the solar panel is characterized by the amplitude of the AC component, *i.e.*, Vpp (peak-to-peak voltage) of the signal relayed by the solar panel.

**Figure 4:**
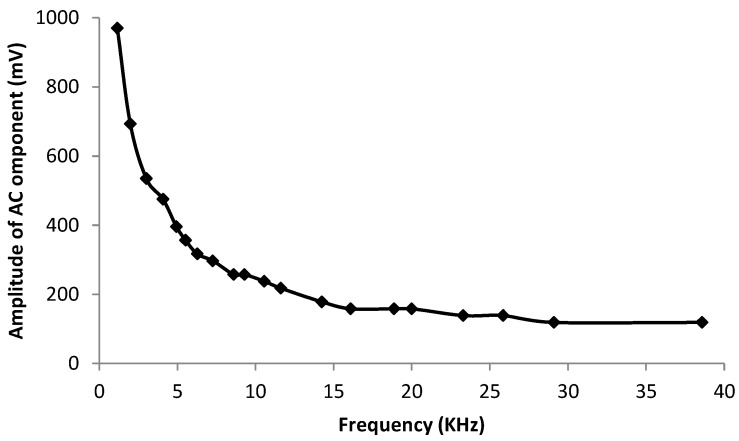
Indoor solar panel response to different frequencies.

As seen in Figure 4, lower frequencies provide higher Vpp values, as also was observed in [4]. In the test, a 10 W LED was used, with a distance of 1 m between LED and the indoor solar panel, and no interference from other sources of light scenario exists. Based on the measured frequency response and the operational frequencies supported by the capabilities of the other receiver components discussed later, we chose to work at 21 kHz. As shown in Section 4, this choice enables the system to work with decent wake-up ranges.

**Figure 5:**
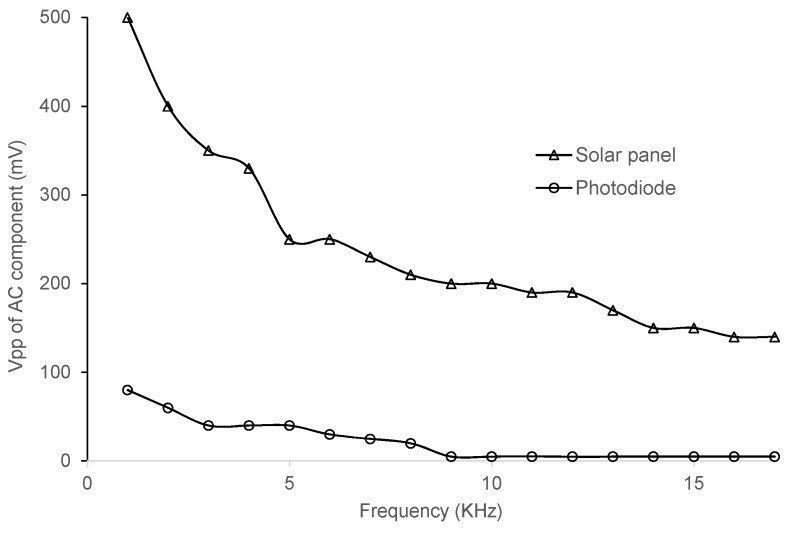
Response of indoor solar panel *vs.* photodiode to a VLC signal modulated with different frequencies.

In the third test, we compare the indoor solar panel to an off-the-shelf high-speed, highly sensitive PIN photodiode. The photodiode selected is the photocell BPW34 (Vishay Semiconductors, Malvern, PA, USA) [11], with a size of 3 mm × 3 mm. In the test we use a separation of 30 cm between the 10 W LED and the photodiode/indoor solar panel. Different frequencies are tested to analyze the frequency response of both options. Figure 5 shows the solar panel and photocell responses to different frequencies. In the graph we can observe that the Vpp of the AC component at the output of the photodiode is lower than that of the indoor solar panel. Starting at a frequency of around 8.6 kHz, the AC component of the signal at the output of the photodiode becomes flat at a value close to 0 mV. Even though photodiodes have a faster response, their achieved Vpp is not high enough for the demodulating signal and the wake-up interruption cannot be generated as proven with further tests. The use of an indoor solar panel also eliminates the need for any amplifier stage after the reception of the signal.

**Figure 6:**
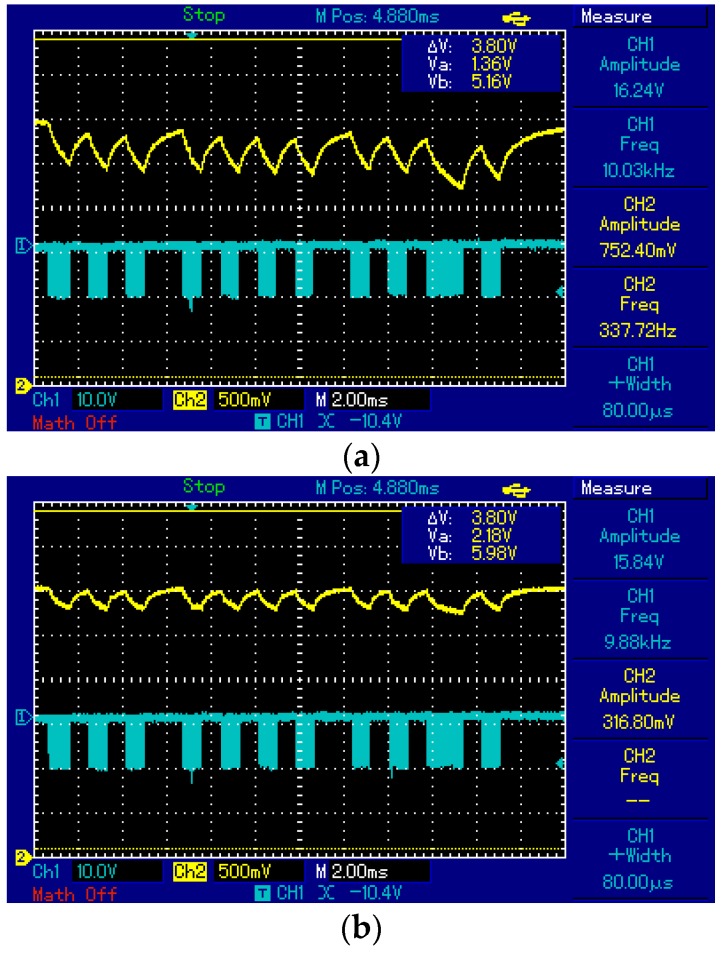
The indoor solar panel responses for (**a**) a small solar panel with dimensions 3.6 × 2.6 × 0.1 cm and (**b**) a large indoor solar panel with dimensions 7.5 × 5.3 × 0.1 cm.

The fourth test compares the indoor solar panel chosen with a larger panel from the same manufacturer, which is also an amorphous silicon solar cell. Figure 6 shows the responses for the small and the large indoor solar panels at a distance of 1.8 m from the LED, and no interferences from external light sources. In the figures, the lower line represents the signal sent and the upper line represents the signal detected by the solar panel. As seen in Figure 6, the Vpp in the case of the large panel is lower than that of the small panel. The reason for this behavior is that the large solar panel maintains a more stable signal level in the output, not relaying the fast variations of light signal amplitude values to the output. Due to this behavior, the maximum distance reached with the large panel is expected to be shorter than the one reached with the small solar panel, which is verified by the experiments presented in Section 4. Note also that, the DC voltage level of large solar panel is slightly higher as expected, although the Vpp values are important in VLC systems for a more successful demodulation.

#### 3.2.2. WuRx Envelope Detector, Demodulator and Correlator

We investigated the off-the-shelf ultra-low power component alternatives for the wake-up receiver parts, that is, for envelope detector, demodulator and correlator. Although many options exist, the total amount of energy consumed by all the individual components exceeds the amount of energy generated by the indoor solar panel. Note that, the power harvested from indoor light is much lower than the solar light, creating a challenge on indoor light harvesting systems. As reference, the indoor solar panel used in our implementation generates the maximum power of 96 μW with a voltage of 3.76 V and a current of 26.6 μA, as shown in Section 3.2.1.

Although ultra-low power components such as the 74VHC595 8-bit shift register [12] which only requires 4 μA as input current and a source voltage of 3 V, are available in the market, to build a wake-up receiver, several other components such as two 8-bit comparators, a rectifier, a Manchester demodulator, a local register for the local ID are required, resulting in a lack of power budget to feed all the needed components. A more power-efficient approach is to utilize an integrated chip that include all these components. After a detailed search, the decision was to use an off-the-shelf ultra-low power, high sensitivity radio wake-up receiver, the AS3933 chip [13] manufactured by AMS, as part of the receiver system. Feeding the electrical signal generated by the solar panel to the antenna input of AS3933 was verified to generate a functional receiver circuit.

AS3933 is internally composed of, aside from other modules, an envelope detector, an OOK demodulator, and a 16-bit correlator as required by the receiver system. The power consumption of this device fits well with the power requirements desired in the project as it requires around 2 μA current consumption in listening mode and around 8 μA in correlation mode working at 2.4–5 V. AS3933 can be configured to work in one of the bands within the possible carrier frequency ranges of between 15 kHz and 150 kHz. We choose to work in the frequency band of 15 kHz to 23 kHz due to the better response of the indoor solar panel to the low frequencies as described in Section 3.2.1.

**Figure 7:**
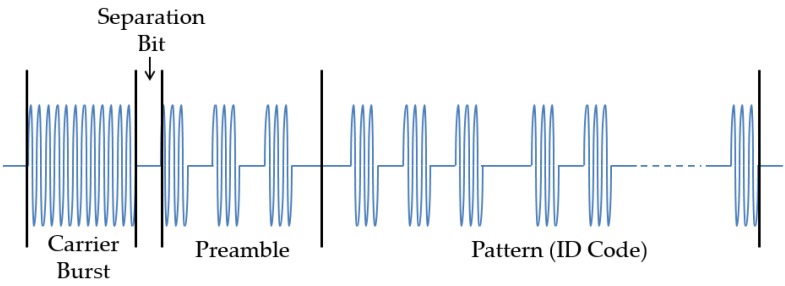
AMS AS3933 wake-up signal format.

AS3933 requires a specific format for wake-up signal, which is depicted in Figure 7. The signal is composed of four parts: Carrier burst, a separation bit with the length of half Manchester symbol, a preamble of six bits (with the 101010 sequence), and the ID code or address of the node intended to be woken up (pattern). In addition to the triggering of a wake-up interrupt based on address match (correlator ON), the chip can also be configured to generate a wake-up interrupt without an address matching, using only the detection of carrier frequency as the trigger of the wake-up interrupt (correlator OFF). This latter feature can be used in applications that require a wake-up of several nodes at the same time, providing a broadcast wake-up feature.

**Figure 8:**
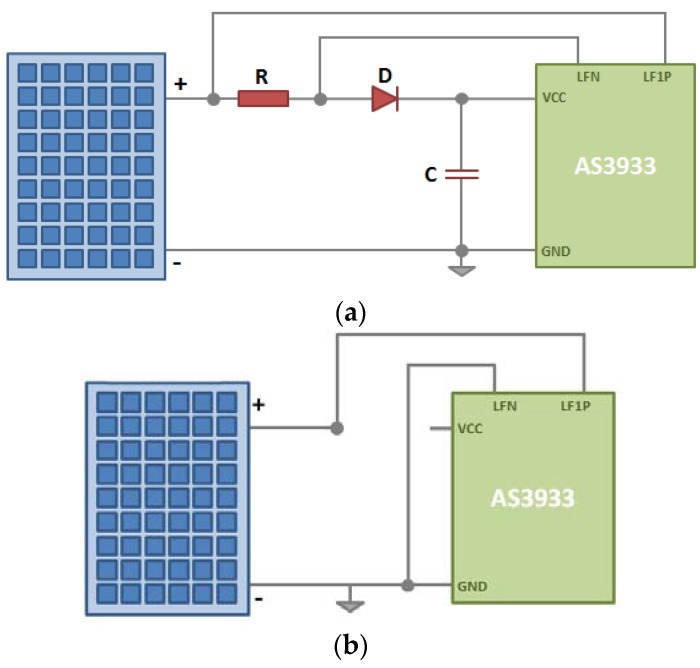
Two possible wake-up receiver circuit design options: (**a**) WuRx design 1 and (**b**) simplified WuRx design 2.

The next step to complete the receiver system is to devise necessary circuitry to connect its components. We deduced two feasible ways to connect the components and complete the WuRx design (Figure 8). In the first circuit design (Figure 8a), a capacitor, C, is used as the storage of the power and is connected directly to the VCC and GND pins of AS3933 chip. A diode, D, is used to prevent the capacitor from discharging through the solar cell and a resistor R is used where a portion of the voltage is used and the variation of the signal is detected. The signal that falls on R is used as incoming signal and is fed to the AS3933 through LF1P and LFN pins, where LF1P is the input of antenna channel 1 and LFN is the ground for the antenna in AS3933 chip [13].

The second WuRx circuit design alternative (Figure 8b) is a simplified version of the first one and would work well if enough illumination is available in the deployment place. In this case no additional components are required, even the capacitor can be removed. This circuit has been tested in controlled indoor scenarios successfully; however, as there is no capacitor to regulate the voltage input to the chip, exposing it to sunlight might damage the AS3933 chip. As an illustrative example, when exposed to sunlight, the indoor solar panel is measured to have a short circuit current of 0.737 mA and an open voltage of 6.07 V, the latter being higher than the AS3933 maximum operational voltage of 5 V. Hence, for the developed system, the first WuRx circuit design option is chosen, considering the potential indirect sunlight reception through windows, *etc.*

**Figure 9:**
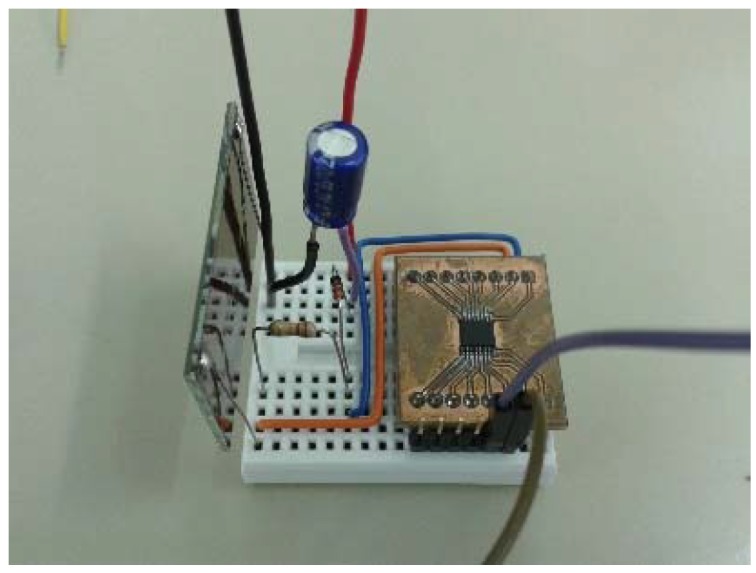
Prototype developed for WuRx design 1.

The choice of the small indoor solar panel and the AS3933 chip components enable small footprint and very low cost (around US $ 4.50) receiver system. The prototype of the receiver system for the WuRx design 1 is pictured in Figure 9.

#### 3.2.3. Wake-Up Transmitter

Unlike the case of the receiver, for the design of the transmitter there are no power restrictions; yet, we opt for a low-cost, small footprint wireless node as the Control Module of the LED, enabling fast and inexpensive integration of the developed system into existing environments. We choose a Z1 device (Zolertia, Barcelona, Spain) for this purpose, due to its low cost and small form factor, while providing support to low power wireless communication protocols such as IEEE 802.15.4 and ZigBee [14].

**Figure 10:**
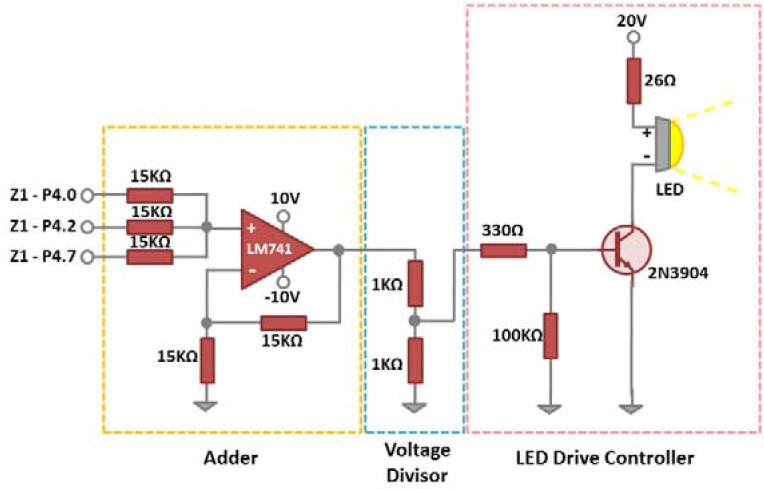
LED drive circuit developed.

For the implementation of the LED drive module, we developed a circuit interfacing the Z1 digital ports, which consists of a summation stage, a voltage divider and a LED drive controller as detailed in Figure 10. We used three digital ports of Zolertia Z1 to generate digital signals and an adder circuit to sum these signals for an Amplitude Shift Keying (ASK) modulation. The values of the resistors in the adder have been chosen to be 15 KΩ to deliver a current value of 0.2 mA from each port in order to protect the Zolertia module (the amount of current exiting from all the output pins of Z1 should not be greater than 1.5 mA [14]). Z1 is able to produce only digital signals with the levels of 0 V and 3 V for low and high logical levels, respectively. Hence, the election of the adder circuit will help us with the generation of ASK signals and different levels of light intensity, which is crucial for developing flickering mitigation approaches as explained in Section 4.

The maximum level of the added signal is 9 V, hence a simple voltage divider is used after the adder circuit, to limit the maximum voltage to 4.5 V. Finally, the LED drive controller uses the 2N3904 NPN transistor for converting the variation of the voltage in its base connection into a proportional variation of the current to flow through the Collector-Emitter connections. The last component of the transmitter is a LED light, for which we opted for a LED light supporting a maximum power of 10 W. The voltage employed (20 V) results in a LED light power consumption of 3.2 W and the LED drive circuit including the Z1 node is measured to consume 87.9 mW.

**Figure 11:**
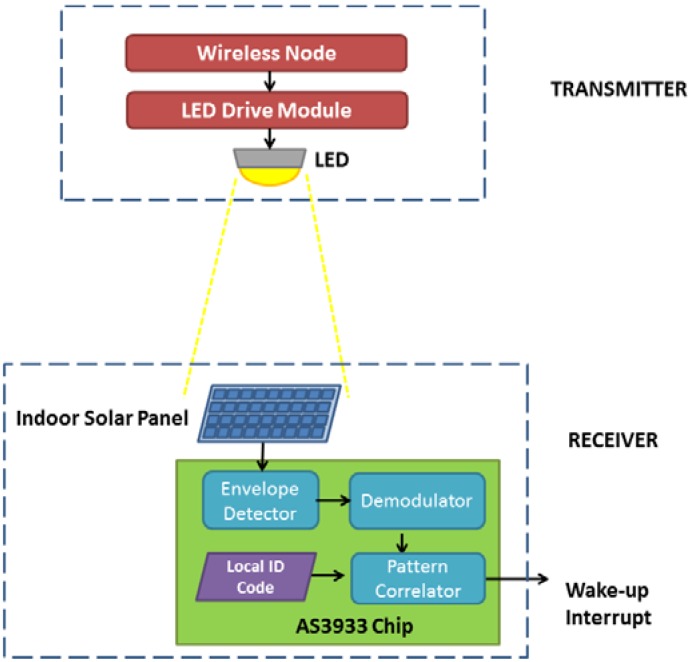
Resulting system view of the solar-panel-based wake-up system.

The resulting solar panel based VLC wake-up communication system is shown in Figure 11. Note that although specific components are chosen for the wireless node and indoor solar panel for our implementation and for the physical experimentations presented in this paper, any off-the-shelf wireless node and indoor solar panel can be used within this system.

## 4. System Evaluations

One of the challenges in Visible Light Communications (VLC) is the flickering, which may appear when the source of light is modulated. Especially, for low data-rate VLC communications, such as for wake-up communication or camera communication, where low carrier frequency is used, this challenge requires careful analysis. Hence, we first investigate the flickering characteristics of the developed system, and propose solutions for its mitigation. Then, we perform operational range evaluations considering different configurations of the system.

### 4.1. Flickering Characterization

When a light source is modulated, flicker may appear. Flickering is the fluctuation in the brightness of light and can become an annoying effect, and develop negative physiological changes in humans [3]. In [15], the Maximum Flickering Time Period (MFTP) is defined to be the maximum time period over which the light intensity can change without being perceived by the human eye. An optimal flickering frequency does not exist, however a frequency greater than 200 Hz (*i.e.*, MFTP < 5 ms) is considered to be safe.

In IEEE 802.15.7 VLC standard, which specifically focuses on high data rate VLC communication, flickering is classified as intra-frame flickering and inter-frame flickering. Intra-frame flickering is the fluctuation in the brightness within a data frame. Mitigation of intra-frame flickering is accomplished by the use of length limiting coding or modulation scheme, e.g., Manchester encoding or Variable Pulse Position Modulation (VPPM) [16,17,18]. On the other hand, inter-frame flickering is the brightness fluctuation between the data frame transmissions. Inter-frame flickering mitigation is accomplished by transmitting an idle pattern between data frames whose average brightness is equal to that of the data frames [16]. In order to mitigate flickering, light dimming can be used [3]. Light dimming consists in the control of the brightness of the light source in order to satisfy a constant brightness.

In order to characterize the flickering the following tests were performed: (i) the first test evaluates the kind of signal generated by the Zolertia Z1 and how it induces flickering; (ii) a second test analyzes how dimming affects the range of the system; (iii) a third test helps us to measure the light brightness generated by each part of the frame; and (iv) finally a fourth test was performed in order to control inter-frame flickering.

The first test was carried out using the Zolertia Z1 for the generation of a *constant* signal of 21 kHz of frequency, which is the carrier frequency chosen. The signal was fed to a LM3409 LED driver module (Texas Instruments, city, state abbrev if USA, country). A flickering appears in the LED, even accomplishing the rule of the use of an MFTP lower than 5 ms (for a signal of 21 kHz of frequency, the period is 0.0047 ms). The test then was repeated generating the signal with a Function Generator with a frequency of 21 kHz. In this second case, no flickering appears in the LED.

In the case of Z1, the flickering is found to be due to the “gaps” introduced by the Z1 into the signal. We conclude that the gaps introduced are due to the fact that Contiki kernel employed in the Z1 does not provide a real-time multi-processing, instead, an event-driven programming is implemented and the processes run in cooperative context, whereas the interrupts and real-time timers run in the preemptive context. In cooperative context the kernel wait for the finalization of the task but in preemptive context the kernel temporarily interrupt the task being carried out in order to execute another task of higher priority level. The interrupted task is resumed at a later time [19]. As the conclusion of this first test, we infer that in order to avoid flickering, a node with an MCU and operating system supporting real-time multi-processing is necessary. One of the future works, hence, will be to investigate such a solution.

One of the methods for flickering mitigation is dimming the LED light. In the second test we generated the same signal with three different levels of dimming with the format of the signal required by the AS3933 (Figure 7). For dimming the signal, we use a simple voltage divider after the output of the Zolertia Z1 pins. The levels of dimming chosen were limited by the characteristics of the Z1. This test was performed using the configuration option 1 of the receiver Figure 8. In the no dimming case, transmitted signal Vpp is 2 V. In the case that the signal was dimmed to have a Vpp of around 1 V, we observe that the flickering decreases slightly, yet, this results in a wake-up range shorter than the one without dimming (detailed operational wake-up range tests are presented in Section 4.2). Finally, with the signal that has a Vpp of approximately 0.6 V, few flickering is perceived in the LED; however no wakeups are triggered at any distance. From this second test, we conclude that even if the flickering is considerably reduced, in order to reach longer distances, the amount of dimming should be limited.

**Figure 12:**
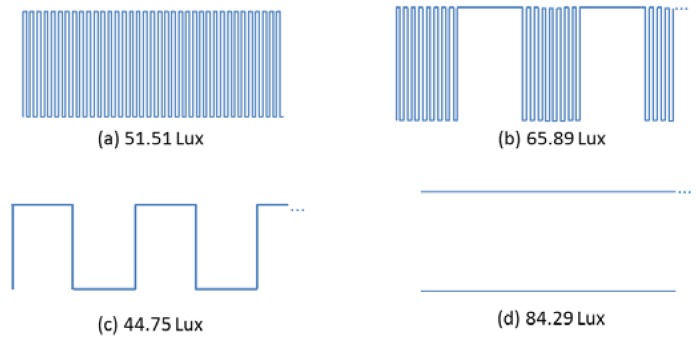
Light intensity measurements for different frame parts of (**a**) carrier burst; (**b**) OOK signal used for preamble and pattern; (**c**) OOK with the data bit rate chosen, and (**d**) for a reference signal of always on.

In the third test, we quantify the light intensity levels generated by different frame components of the format shown in Figure 7, using a light meter. The signals tested are one with a frequency of 21 kHz (Figure 12a), one with OOK modulation with bit duration defined in this study (Figure 12b) and a frequency of 2.625 kHz which corresponds to the bit rate (Figure 12c). As point of reference the intensity of light was measured when the signal is not modulated, which is achieved by feeding the LED with a constant DC signal (Figure 12d). The measurements are done at a distance of 1 m and with no interference from other sources of light.

As seen in Figure 12, different modulations yield different light intensities, which might result in flickering. The OOK modulated signal according to the bit duration of the system is the one with the highest illuminance level among the modulated signals. This can be explained by the fact that in the OOK modulated signal, half of the time the light remains in the higher level of intensity, as we choose to keep the LED on for the data corresponding to the logical zero. The reason is that the use of high voltage level for logical zero allows the lights to be on during most of time, reducing flickering and increasing the intensity level of the light, and as a result an increased operational range. Note that the carrier burst of the frame corresponding to the signal in Figure 12a provides a different intensity level than the signal used for the preamble and pattern shown in Figure 12b.

**Figure 13:**
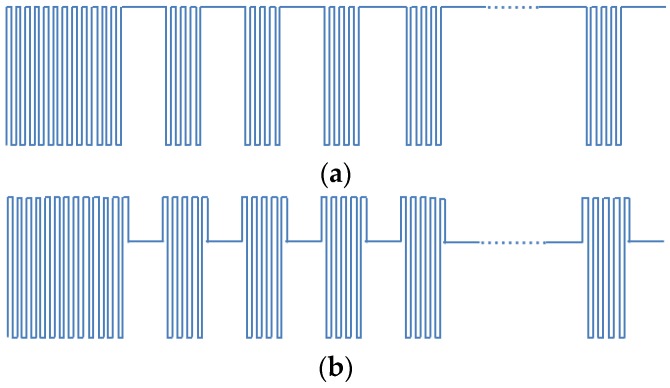
Intra-frame flickering compensation. (**a**) Signal without dimming; (**b**) Signal with dimming applied to the logic zero bits.

As stated before, the flickering can be categorized as intra-frame flickering and inter-frame flickering. For intra-frame flickering, splitting the frame in sub-frames and add some “compensation bits” between them is recommended [3]. The compensation bits will help to maintain the same brightness during the transmission of the frame. In the case of our implementation, it is not possible to add compensation bits inside the frame, since the requirements of the frame format of AS3933 cannot be changed. Instead, in our implementation, we propose to dim the signal in certain points, helping to maintain the level of brightness in the LED. In Figure 13, the signal using the two formats is shown: Figure 13a shows the signal without dimming and Figure 13b presents our dimming solution applying a compensation by adjusting the signal level of the logic zero data bits. Also the use of Manchester encoding is recommended for intra-frame flickering mitigation [3], and implemented in the proposed frame shown in Figure 13.

**Figure 14:**
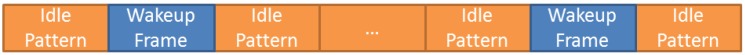
Complete frame format of VLC communication.

For inter-frame flickering compensation, it is necessary to introduce an idle pattern between frames. It is important to choose an idle pattern with a similar level of brightness with the frame. As a trivial solution, the idle pattern is defined to use the same format with the data frame. That is, the idle pattern is also composed of a carrier burst, separation bit, preamble bits and pattern bits (or wake-up code), using a special wake-up code in the pattern. The full stream is then built following the format shown in Figure 14, where the wake-up frames are included between the streams of 50 idle patterns.

As the fourth test, measurements of the amount of light intensity generated by this frame stream are performed. For the generation of the frame we used: (i) a data stream using frames without dimming (Figure 14a) and (ii) a data stream using frames with dimming in the logic zero bits (Figure 14b). The results are shown in Table 2. Again a distance of 1 m between the LED and the light intensity meter was established and no interference by external sources of light exist.

As can be observed in the results presented in Table 2, in the case (ii) the variation in illuminance is 7 lux less than the one in case (i). Also, in the case (i), the illuminance range varies between the values reached by a signal of 21 kHz (Figure 13a) and a signal modulated using OOK with bit duration of the system (Figure 13b). In case (ii) the intensity levels are closer to the ones reached by the carrier burst signal of 21 kHz, which provides a more stable signal in terms of light intensity.

It is important to mention that different inter-frame, *i.e.*, idle patterns were also tested (*i.e.*, Manchester symbols, frequency of 21 kHz, frequency of 2.65 kHz among others, all with different levels of dimming). However, none of them mitigates flickering as much as the approach presented in Figure 13b. Based on the results of this test we decided to dim the signal in zero logic bits, use Manchester coding for intra-frame flickering mitigation, and use the idle pattern described for inter-frame flickering mitigation in Figure 13b. The dimming in the signal is applied to the wake-up frame and also to the idle pattern.

**Figure 15:**
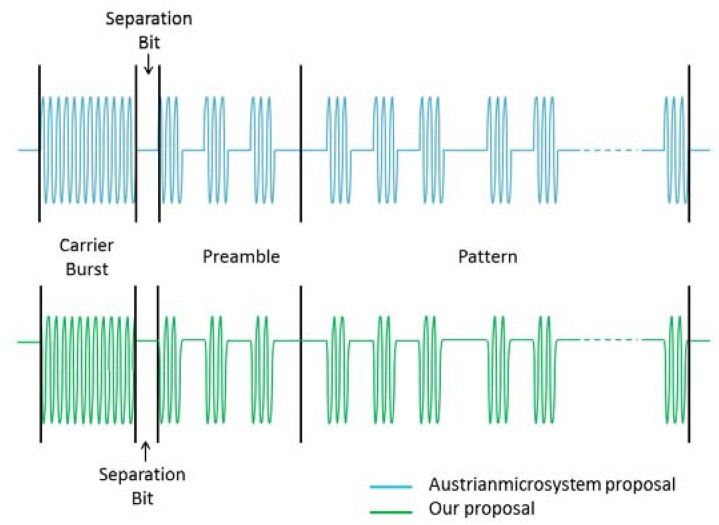
Transmitted signal format: AMS proposal *vs.* our proposal.

After this set of tests, the format of the wake-up signal derived is shown in Figure 15. For the generation of the signal, three ports (P4.0, P4.2 and P4.7) of the Zolertia Z1 are used. The objective of using three ports is the possibility of creating a signal of three levels of intensity of brightness. Note that with the Zolertia Z1 ports it is possible to generate only rectangular signals putting the pins in high or low level.

In Figure 15, both the AMS proposal for the signal and our proposal are depicted. We propose a change in the way the signal is sent: as we are working with light, the default state (or zero logic bits) of the signal should have a “higher level” than the one proposed by AMS. With this small change the lights are in “on” state for longer, which brings several advantages such as the reduction of flickering, having more light to be harvested by the indoor solar panel without any change in the AS3933 behavior.

### 4.2. Wake-Up Range Evaluations

For a wake-up communication system, the wake-up range determines the applications that can be supported by the developed system. In this section, we depict the wake-up range evaluations of the base system configuration, then propose and evaluate alternative settings to the base system configuration to improve the system performance.

**Figure 16:**
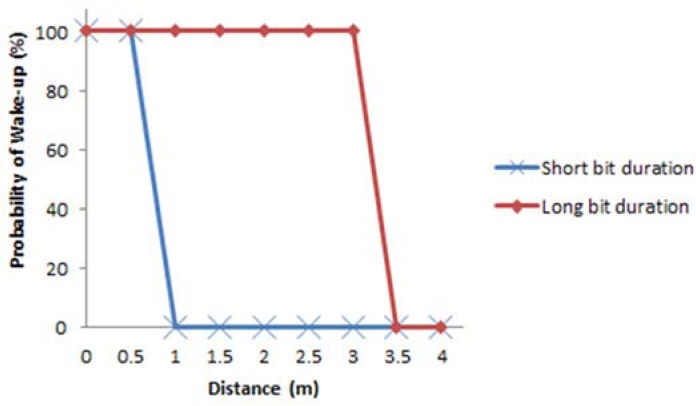
Probability of wake-up *vs.* distance for short and long bit duration, under interference from fluorescent light with correlator setting ON.

In the first set of tests, we use the signal format proposed in Figure 15 to evaluate the wake-up probabilities per distance in presence of interference from other light sources (office fluorescent lights used in this test as interference). The main evaluation environment used is an office/laboratory with a size of 10 × 4 × 2.4 m (L × W ×H) that includes desks, bookshelves and electronic equipment. The WuTx is fixed on a desk and the WuRx is displaced within the evaluation environment for the range evaluations. For the calculation of the wake-up probability, for each evaluated distance, we send the signal 50 times and we count the number of times the wake-up signal was triggered. The range evaluations are done with bit duration of 381.264 μs which is the value finally chosen for this implementation; however the range evaluations with bit duration of 755.786 μs are also presented for comparison. Different bit durations are assessed for two important issues: With shorter bit duration the flickering of the light source is lower, and with shorter bit duration it is possible to reach a higher transmission bit rate. The configuration used for the receiver is the one shown in Figure 8 and the frame structure and the signal formats are the ones in Figure 14 and Figure 15, respectively. In this case a full signal (carrier burst + separation bit + preamble + pattern) was sent, in other words, we set the internal correlator setting of AS3933 to ON to wake up only one targeted device. The results are depicted in Figure 16.

With the short bit duration a maximum distance of 0.6 m was reached and with the longer bit duration the maximum distance is found to be 3.1 m. The further distance reached with the longer bit duration is due to the fact that each bit has a longer exposure and consequently a higher brightness in the source of light, increasing the probability of successful demodulation. Note that the wake-up probabilities fall down sharply once the operational distance limits are reached. The negative impact of interference on the performance can be explained by the solar panel’s capacitive behavior resulting in slow responses to fast signal level changes caused by interference.

The number of signal level changes are also increased further with the short bit duration setting, resulting in a limited wake-up distance performance. Given the increased flickering caused by longer bit durations due to the higher intra-frame brightness differences, we choose the shorter bit duration and focus to improve the system range for this setting.

**Figure 17:**
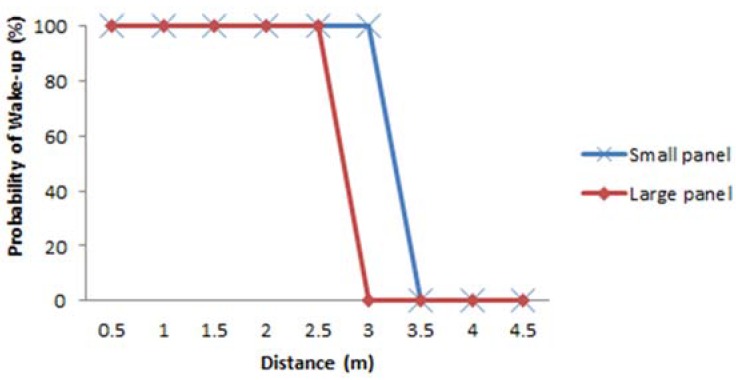
Probability of wake-up *vs.* distance for small and large indoor solar panels, under interference from fluorescent light with correlator setting ON.

The next set of tests are performed to observe the effect of the solar panel size on the wake-up probabilities, evaluating the distances reached by large and small solar panels. The results are shown in Figure 17, where we observe that the maximum distance reached with fluorescent light interference is 2.5 m for the larger panel and 3 m for the small panel. The reason for this behavior is that large solar panel maintains a more stable signal level in the output, not relaying the fast variations of light signal amplitude values to the output, as also illustrated in Figure 6.

**Figure 18:**
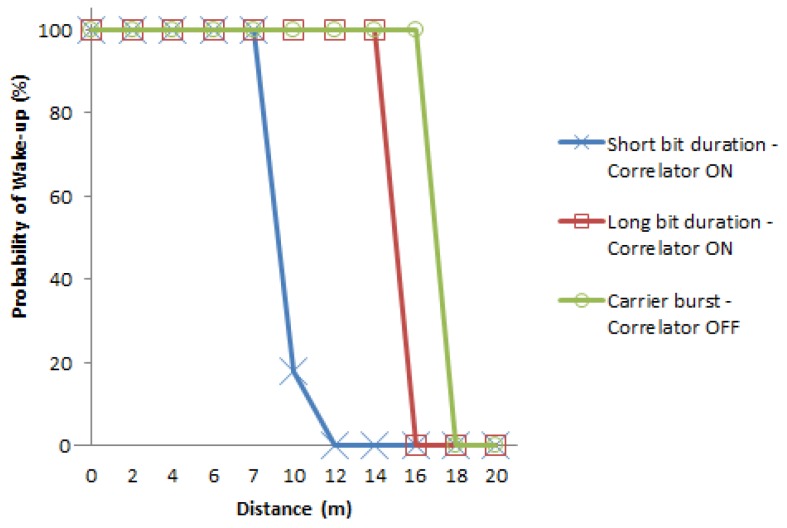
Probability of wake-up *vs.* distance for the correlator ON and OFF settings, for short and long bit duration and under no interference scenario.

The third set of tests are done to evaluate the wake-up distances for the case of no interference and using the two possible configurations for the transmitted signal. Note that this setup corresponds to an operational indoor environment, where the interference can be controlled, such as when the lighting system consists of coordinated LEDs. In the first configuration a signal with carrier burst, separation bit, preamble and pattern is sent (correlator ON), using the two different bit durations considered. We identify the probability of the successful wake-up of the signal *versus* the distance from transmitter to receiver. In a similar way, in the second configuration only a carrier burst with frequency of 21 kHz was sent, *i.e.*, with the correlator OFF. A very large seminar room is used for this set of evaluations to allow the experimentation of a wide range of distances. The results are shown in Figure 18. With the correlator ON, we reach a distance of 14 m for the case of long bit duration, and 7 m for the case of short bit duration; and with the correlator OFF and using a burst carrier with duration of 300 carrier frequency periods, the maximum distance achieved was 15.5 m. As it was expected, the distance reached with the correlator OFF is longer because the AS3933 only has to identify the presence of a carrier frequency of 21 kHz and is not affected by the bit duration or the format of the frame. Again we observe that the wake-up probability falls down quickly once the limited distance is reached.

The flickering in the LED is lower for the case of short bit duration than for the case of long bit duration. In the case of short bit duration, the perception of flickering is mainly noticed near the LED. In both cases, using the solar panel to power AS3933 *versus* using the USB for the same purpose, the distances achieved were the same.

A further test with the correlator setting OFF and with fluorescent light interference in the office environment is also performed. In this case, a high number of false wake-ups is observed due to the optical noise of the test environment. This shows how important it is to use the correlator in order to avoid any undesirable behavior of the system.

**Figure 19:**
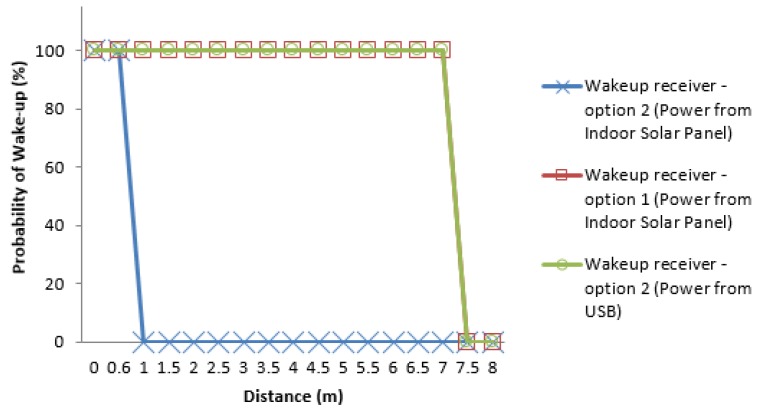
Probability of wake-up *vs.* distance for the correlator ON for the different receiver configuration under no interference scenario.

A final test was developed using the WuRx design options 1 and 2, presented in Figure 8. The test was done in the office environment without light interference. In the case of receiver design option 1, once the capacitor is charged the maximum distance reached is also 7 m. Using the receiver design option 2, a maximum distance of 60 cm was reached; however using the same configuration with the receiver being fed with USB as power source, a distance of 7 m was reached. Figure 19 shows the result of this test.

The short distance reached with the receiver design option 2 is easily explained by the fact that with the indoor solar panel it is not possible to harvest enough power for the operation of the AS3933 from the amount of light emitted by this single LED at longer distances. Hence, using a capacitor and storing the energy (WuRx option 1) is necessary for the battery-less execution of the proposed VLC-based wake-up system. Based on these test results, it is clear that the distances achieved by the proposed system are enough for many indoor applications, such as asset control, tracking of devices, security systems, and others.

## 5. Conclusions and Future Work

A wake-up receiver system that uses VLC for communication and an indoor solar panel for energy harvesting and reception of the VLC signal has been presented. The use of VLC for communication avoids the problem of radio interferences with other communication signals and preventing the congestion in the radio channels. Also, the wake-up receiver enables an on-demand communication system avoiding the unnecessary wake-up of the wireless sensor nodes attached. In the tests performed a wake-up distance of 7 m is reached in case of no external interference, which is a targeted distance for VLC systems. However using longer bit durations, it is possible to reach longer distances of around 14 m, which exceeds the targeted range of distance.

The system also has the novelty of the use of an indoor solar panel as the VLC signal receiver, allowing the use of the indoor solar panel for a different purpose than the one they were built for. Also, the use of an indoor solar panel permits harvesting the necessary power from the light for the energy-autonomous operation of the wake-up receiver. This allows the wake-up receiver to be in constant and independent activity without affecting the battery lifetime of the wireless sensor node, saving in this way significant power and consequently extending the lifetime of the sensor device connected to wake up receiver and the network.

Also the addressable wake-up feature of the developed system enables on-demand wake-up of only the targeted node. Nevertheless, it was shown that the wake-up receiver can also generate a wake-up signal using the carrier burst, which can be useful in environments where it is desirable to wake up several sensor nodes at once.

Using an indoor solar panel in the system also brings a set of new issues and challenges, for example, the restriction in the use of high frequencies for the transmission and consequently lower bit rates; and the use of ultra-low power components for the receiver due to limited energy harvested from indoor lighting systems. In addition, the tests performed using a small and a large size indoor solar panel show a better frequency response for the small one, which permits the use of the system in applications where the small size of the device is a desirable characteristic.

Flickering mitigation is still a challenge that must be investigated further. In this study, the flickering was minimized through several actions: Intra-frame flickering mitigation, inter-frame flickering mitigation, dimming of the signal in the zero logic bits, the use of a shorter bit duration time and the reduction of the length of the signal sent.

It is important to mention that upon the triggering of the wake up signal, it is possible to transmit data using the VLC channel for downlink data communication, which is a future work topic. Also, the study of flickering mitigation in VLC communications can be furthered, along with the developing of a transmitter with more levels of dimming, and the possibility of inserting compensation bits within the frame. Another future work involves the detailed study of the use of capacitors to accumulate the energy harvested by the solar panel for a longer wake-up range.

## Figures and Tables

**Table 1:** An Illustrative Comparison of Different Wake-up Communication Mode Studies.

	[9]	[6]	[7]	[8]	This Work
Comm. Mode	RF	Infrared	VLC	Ultrasound	VLC
**Power Source at Receiver**	Sensor’s battery	Coin cell battery	Sensor’s battery	Sensor´s battery	Power harvested from light
**Receiver Power**	22.9 μW	12 μW	695 pW	4.8 μW	~95 μW
**Transmitter Power**	N/A	~60 mW (IR remote control)	3.5 mW (Only laser, controller power N/A)	16 μW	87.9 mW (Only LED controller)
**Max Data Rate**	200 Kbps	N/A	91 bps	250 bps	1.12 Kbps
**Range**	−75 dBm (Sensitivity)	30 m	50 m	8.6 m	14 m
**Requirements**	Battery required for the receiver	Limited interference	Highly directional link for laser	Other energy harvesting source	Targeted for indoor systems

**Table 2:** Illumination Level of the Frame Stream Using Different Levels of Dimming.

No.	Description	Lux
Case (i)	Data stream (Figure 14) using frames without dimming (Figure 14a)	47.5–65.5
Case (ii)	Data stream (Figure 14) using frames with dimming in the logic zero bits (Figure 14b)	45.5–53.5
